# Supplementary material for: Effects of thymidylate synthase inhibitors differ in genomic uracilation and mutagenic potential
Source: Life Sci Alliance. 2026 Feb 6;9(4):e202503352. doi: 10.26508/lsa.202503352 (PMC12881662; doi:10.26508/lsa.202503352)
Supplement: Supplementary file 5 [file LSA-2025-03352_Supplemental_Data_1.docx]

**#Genome segmentation (Fig 1 and Fig S1A)**; Coverage ratios of enriched and input samples using deepTools package (version 3.2.1), (cf. GEO Series GSE126822). The ratio.bdg files were filtered for the core chromosomes and used in genome segmentation analysis by Segway.

bigwigCompare -b1 NAME_enriched.bin100bp.smooth5000.RPGC.bw -b2 NAME_input.bin100bp.smooth5000.RPGC.bw -o NAME.bin100bp.smooth5000.RPGC.ratio.bdg --operation ratio -of bedgraph --binSize 100 --skipNAs -v -p 32

awk '($1=="chr1") || ($1=="chr2") || ($1=="chr3") || ($1=="chr4") || ($1=="chr5") || ($1=="chr6") || ($1=="chr7") || ($1=="chr8") || ($1=="chr9") || ($1=="chr10") || ($1=="chr11") || ($1=="chr12") || ($1=="chr13") || ($1=="chr14") || ($1=="chr15") || ($1=="chr16") || ($1=="chr17") || ($1=="chr18") || ($1=="chr19") || ($1=="chr20") || ($1=="chr21") || ($1=="chr22") || ($1=="chrX") || ($1=="chrY") {print $0}' NAME.ratio.bdg > NAME.ratio.filtered.bdg

genomedata-load -t {SAMPLE1}={FILE1} -t {SAMPLE2}={FILE2} … -s core_regions.fna mergedload

SEGWAY_CLUSTER=local segway train mergedload --resolution=100 --num-labels=12 --minibatch-fraction=0.01 --exclude-coords=blacklist_merged_d500.bed train12_merged_blacklisted

SEGWAY_CLUSTER=local segway annotate mergedload train12_merged_blacklisted Segway_annot_out

segtools-signal-distribution segway.bed mergedload

segtools-aggregation --normalize segway.bed gencode34.gff

segtools-length-distribution segway.bed

#Heatmap (Fig 1 and Fig S1A) generation by Seaborn (MatPlotLib module of Python):

from matplotlib import pyplot as plt

from matplotlib import patches

import pandas as pd

import seaborn as sns

df = pd.read_csv("signal_blacklisted.tab",*sep*="\t")

df2 = df[["label","trackname","mean"]]

df2 = df2.pivot_table(*index*='trackname', *columns*='label', *values*='mean')

df2.columns = [col for col in df2.columns]

df3 = df[["label","trackname","sd"]]

df3 = df3.pivot_table(*index*='trackname', *columns*='label', *values*='sd')

df3.columns = [col for col in df3.columns]

# normalization

df4 = (df2 - df2.min().min())/(df2.max().max()-df2.min().min()) # normalize means

fig = plt.figure()

ax = fig.add_subplot(111)

ax = sns.heatmap(df4,*cmap*="RdYlBu_r")       #draw means

plt.tick_params(*axis*='both', *which*='major', *labelsize*=20, *labelbottom* = False, *bottom*=False, *top* = False, *labeltop*=True)

ax.tick_params(*length*=0)

for i in range(df2.shape[0]):

    for j in range(df2.shape[1]):

        bottom_left_x = j + (1-df2.iloc[i,j]/4)/2

        bottom_left_y = i + 0.25

        # Create a rectangle (smaller square)

        rect = patches.Rectangle(

            (bottom_left_x, bottom_left_y),

            df2.iloc[i,j]/4, 0.5,

*linewidth*=2,

*edgecolor*='black',

*facecolor*='black'

        )

        # Add the rectangle to the plot

        ax.add_patch(rect)

        #ax.add_patch(patches.Rectangle((x - df2.iloc[i,j]/4,y),df2.iloc[i,j]/2,0.5,facecolor="black",edgecolor="black"))

plt.show()

# Calculation of bedtools Jaccard indices between the segment labels (Fig S1B).

import pandas as pd

df = pd.read_csv("segway.bed", delimiter="\t")

for label in range(12):

df_out = df[df[“label”] == label]

df_out.to_csv(f”segway_{label}.bed, index = False, sep=”\t”)

bedtools jaccard -a segway_merged_label.bed -b segway_reps_label.bed

#Heatmap (Fig S1B) using Seaborn module.

import pandas as pd

df = pd.read_csv("jaccard_table.txt", delimiter="\t")

s=sb.heatmap(df,xticklabels=True,yticklabels=True,linewidths=0.5,linecolor="black",cmap="RdYlBu_r",annot=False,annot_kws={'size':4})

figure = s.get_figure()

figure.savefig('plot_jaccard.png', dpi=400)

**#Calculation of replication timing score and AT content for the genomic segments (Fig 1 and Fig S1A)**

#Two datasets (Int90617792 and Int97243322) in Replication Domain Database were used to calculate the average replication timing scores.

awk '($2=$2-1) {print $1 "\t" $2 "\t" $3 "\t" $4}' Int90617792.bed | sort -k1,1 -k2,2n > Int90617792_0start.bed

awk '($2=$2-1) {print $1 "\t" $2 "\t" $3 "\t" $4}' Int97243322.bed | sort -k1,1 -k2,2n > Int97243322_0start.bed

awk '($2=$2-1) {print $1 "\t" $2 "\t" $3 "\t" $4}' RT_HCT116_merged.bed | sort -k1,1 -k2,2n > RT_HCT116_merged_0start.bed

#Example for the separation of the segments into distinct bed files:

awk '$4==0 {print $0}' segway.bed | sort -k1,1 -k2,2n | awk '{print $1 "\t" $2 "\t" $3 "\t" NR}' > segway_merged12.seg0.bed

#Example for taking the intersections of bed files defining the RT scores and the genomic segments:

bedtools intersect -wo -a segway_merged12.seg0.bed -b Int90617792_0start.bed > RTscore.segway_merged12.seg0.intersect.bed

#Example for calculating the average RT score for the prepared bed files:

awk '{print $1 "\t" $2 "\t" $3 "\t" NR}' RTscore.segway_merged12.seg0.intersect.bed > segway.0.RTintersect.ready.bed

bigWigAverageOverBed -bedOut=segway.0.RTscoreAverage.bed Int90617792.bw segway.0.RTintersect.ready.bed DEL.tab

awk '{($6=$3-$2)}1' segway.0.RTscoreAverage.bed > segway.0.RTscoreAverage.length.bed

awk '{($7=$5*$6)}1' segway.0.RTscoreAverage.length.bed > segway.0.RTscoreAverage.averBase.bed

awk '{(sum1 += $7) (sum2 += $6)} END {print (sum1/sum2)}' segway.0.RTscoreAverage.averBase.bed

#Example for the calculation of the AT content:

awk '{print $1 "\t" $2 "\t" $3 "\t" NR}' segway_merged12.seg0.bed > segway_merged12.seg0.ready.bed

bedtools nuc -fi refGenome_core_regions.fna -bed segway_merged12.seg0.ready.bed > segway_merged12.seg0.nuc.bed

awk '{(sum1+=$7) (sum2+=$10) (sum3+=$8) (sum4+=$9) (sum5+=$11) (sum6+=$12) (sum7+=$13)} END {print sum1 "\t" sum2"\t" sum3 "\t" sum4 "\t" sum5 "\t" sum6 "\t" sum7}' segway_merged12.seg0.nuc.bed >> segway_merged12.AT_cont_nuc.scoreSumma.csv

**#Calculation of gene-specific U-scores (Fig 2A)**

#Genome-scaled coverage tracks by bamCoverage, and the enriched/input ratio.bdg files by bigWigCompare of the deeptools package (version 3.2.1.). p-tracks calculated to 100 nt windows, excluding blacklisted regions. U-score for genes listed in GencodeV34, including 1000 nt upstream region and introns.

bigwigCompare -b1 NAME_enriched.bin100bp.smooth5000.RPGC.bw -b2 NAME_input.bin100bp.smooth5000.RPGC.bw -o NAME.bin100bp.smooth5000.RPGC.ratio.bdg --operation ratio -of bedgraph --binSize 100 -v -p 32

bedtools intersect -v -a NAME.ratio.bdg -b common_blacklist_and_masked_merged_d1000.bed > NAME.ratio.cleaned.bdg

#Calculation of p-tracks (uracil / million bases) considering *total uracil content* listed in Supplementary Table 1.

awk 'NR==FNR{sum+=($3-$2)*$4; next} {print $1, $2, $3, ($4/sum)*total_uracil_content*1000000}' NAME.ratio.cleaned.bdg NAME.ratio.cleaned.bdg > NAME.p-trackM.bdg

bedGraphToBigWig NAME.p-trackM.bdg GRCh38.d1.vd1.chrom.sizes NAME.p-trackM.bdg.bw

#Preparation of gene list bed files

awk '{print $2 "\t" $4 "\t" $5"\t" $1 "\t" $3 "\t" $16 "\t" $17 "\t" $18 "\t" $24 "\t" $25 "\t" $26}' wgEncodeGencodeBasicV34_selected > GencodeV34_ENST_UNIPROT.bed

awk '($5=="+") {print $0}' GencodeV34_ENST_UNIPROT.bed > GencodeV34_ENST_UNIPROT_plusSTR.bed

awk '($5=="-") {print $0}' GencodeV34_ENST_UNIPROT.bed > GencodeV34_ENST_UNIPROT_minusSTR.bed

awk '{print $1 "\t" ($2-1000) "\t" $3 "\t" $4 "\t" $5 "\t" $6 "\t" $7 "\t" $8 "\t" $9 "\t" $10 "\t" $11}' GencodeV34_ENST_UNIPROT_plusSTR.bed > GencodeV34_ENST_UNIPROT_plusSTR_with1kPROMOTER.bed

awk '{print $1 "\t" $2 "\t" ($3+1000) "\t" $4 "\t" $5 "\t" $6 "\t" $7 "\t" $8 "\t" $9 "\t" $10 "\t" $11}' GencodeV34_ENST_UNIPROT_minusSTR.bed > GencodeV34_ENST_UNIPROT_minusSTR_with1kPROMOTER.bed

cat GencodeV34_ENST_UNIPROT_minusSTR_with1kPROMOTER.bed GencodeV34_ENST_UNIPROT_plusSTR_with1kPROMOTER.bed | sort -k1,1 -k2,2n > GencodeV34_ENST_UNIPROT_with1kPROMOTER.bed

bedtools intersect –wa -a GencodeV34_ENST_UNIPROT_with1kPROMOTER.bed –b GRCh38.d1.vd1.chrom.sizes.bed | awk '{print NR "\t" $1 "\t" $2 "\t" $3 "\t" $4 "\t" $5 "\t" $6 "\t" $7 "\t" $8 "\t" $9 "\t" $10 "\t" $11}' GencodeV34_ENST_UNIPROT_with1kPROMOTER_NR_intersected_formated.csv

awk '{print $2 "\t" $3 "\t" $4 "\t" $1}' GencodeV34_ENST_UNIPROT_with1kPROMOTER_NRcr_intersected_formated.csv > GencodeV34_ENST_UNIPROT_with1kPROMOTER_usablecol3NR.bed

#Average uracil enrichments using bigWigAverageOverBed.

bigWigAverageOverBed -bedOut=NAME.TRANSCRIPT_list_aver_U-DNA_p-value.bed NAME.p-trackM.bdg.bw GencodeV34_ENST_UNIPROT_with1kPROMOTER_usablecol3NR.bed DEL.tab

awk '{printf "%d\t", $4; printf "%s\t", $1; printf "%d\t", $2; printf "%d\t", $3; printf "%f\n", $5}' NAME.TRANSCRIPT_list_aver_U-DNA_p-value.bed | sort -k1,1n > NAME.TRANSCRIPT_list_aver_U-DNA_p-value.formated.csv

paste -d" " NAME.TRANSCRIPT_list_aver_U-DNA_p-value.formated.csv GencodeV34_ENST_UNIPROT_with1kPROMOTER_NRcr_intersected_formated.csv | awk '{print $2 "\t" $3 "\t" $4 "\t" $1 "\t" $5 "\t" $10 "\t" $11 "\t" $12 "\t" $13 "\t" $14 "\t" $15 "\t" $16 "\t" $17}' > NAME.TRANSCRIPT_list_aver_U-DNA_p-value.annot.bed

#Calculating the number of bases that are overlapping with the blacklist:

bedtools intersect -wo -a NAME.TRANSCRIPT_list_aver_U-DNA_p-value.annot.bed -b common_blacklist_and_masked_merged_d1000.bed > NAME.TRANSCRIPT_list_aver_U-DNA_p-value.annot.overlapBP.bed

#Summing the overlapping bases belonging to the same transcript (awk script):

awk '

NR==FNR{

ID[NR]=$13

overlapBP[NR]=$17

j = 1

next

}

{

sumOverlapBP[FNR] = 0

while (ID[j] == $13) {

sumOverlapBP[FNR] += overlapBP[j];

j = j + 1;

}

{OFS = "\t"}; {print $0, sumOverlapBP[FNR]};

}

' NAME.TRANSCRIPT_list_aver_U-DNA_p-value.annot.overlapBP.bed NAME.TRANSCRIPT_list_aver_U-DNA_p-value.annot.bed > NAME.TRANSCRIPT_list_aver_U-DNA_p-value.annot.sumOverlapBP.bed

#Filtering the fully blacklisted genes and calculating the proportion of blacklisted regions of the rest of the genes. The final lists consist of 89969 gene isoforms in each case.

awk '($14<(($3-$2))) {print $0 "\t" $14/($3-$2)}' NAME.TRANSCRIPT_list_aver_U-DNA_p-value.annot.sumOverlapBP.bed > NAME.TRANSCRIPT_list_aver_U-DNA_p-value.final_cleaned.bed

#Eliminating redundancy by taking the longest isoform of each gene (unique Ensembl gene ID). The non-redundant lists consist of 44126 genes in each case.

awk '{print $0 "\t" ($3-$2) "\t" $8}' NAME.TRANSCRIPT_list_aver_U-DNA_p-value.final_cleaned.bed | sort -k17,17 -k16,16nr | uniq -f16 > NAME.TRANSCRIPT_list_aver_U-DNA_p-value.final_cleaned.nonRedundant_longest.bed

#Selection of U-scores for non-redundant protein-coding gene list (19479 genes in each case)

awk '($10=="protein_coding") {print $9 "\t" $5}' NAME.TRANSCRIPT_list_aver_U-DNA_p-value.final_cleaned.nonRedundant_longest.bed | sort -k2,2nr > NAME.hierarchic_list_U-score_forSTRING.txt

#Calculating the number of genes that belong to the different categories in the top 2000 highest U-score genes (Fig 2B)

awk '{print $0 "\t" $8}' NAME.TRANSCRIPT_list_aver_U-DNA_p-value.final_cleaned.nonRedundant_longest.bed | sort -k5,5nr | head -n2000 | awk '{print $10}' | sort | uniq -c >> top2000Uscore_distribution_longest_isoform.csv

**#Calculation of differential uracilation and creating volcano plots (Fig 2C) using ggplot2 in R.**

#Calculation of the relative U-scores:

merged1 <- read.csv("RTX_UGI_vs_5FdUR_UGI.Uscore_table.reps.csv",sep="\t",header=TRUE)

meanFdUR1 <- mean((merged1$FdUR_rep1_u_score_raw + merged1$FdUR_rep2_u_score_raw)/2)

meanRTX1 <- mean((merged1$RTX_rep1_u_score_raw + merged1$RTX_rep2_u_score_raw)/2)

merged1$RTX_rep1_uracil_score_norm <- merged1$RTX_rep1_u_score_raw/meanRTX1

merged1$RTX_rep2_uracil_score_norm <- merged1$RTX_rep2_u_score_raw/meanRTX1

merged1$FdUR_rep1_uracil_score_norm <- merged1$FdUR_rep1_u_score_raw/meanFdUR1

merged1$FdUR_rep2_uracil_score_norm <- merged1$FdUR_rep2_u_score_raw/meanFdUR1

#Calculation of the log_2_(fold change) and the p-values:

merged1$log_fold_change <- log2((merged1$FdUR_rep1_uracil_score_norm+merged1$FdUR_rep2_uracil_score_norm)/(merged1$RTX_rep1_uracil_score_norm+merged1$RTX_rep2_uracil_score_norm))

p_values <- vector(mode="numeric", length=nrow(merged1))

for (i in 1:nrow(merged1))

{

p_values[i] <- t.test(x = c(merged1$RTX_rep1_uracil_score_norm[i], merged1$RTX_rep2_uracil_score_norm[i]), y = c(merged1$FdUR_rep1_uracil_score_norm[i], merged1$FdUR_rep2_uracil_score_norm[i]))$p.value

}

merged1$p_values = p_values

merged1$log_p_values = -1*log10(merged1$p_values)

merged1$Significant <- ifelse(merged1$log_p_values > 1.30103 & abs(merged1$log_fold_change) > 0.58496 , "Significant", "Not Significant")

write.table(merged1, file="RTX_UGI_vs_5FdUR_UGI.Uscore.volcano.csv")

#Selection of protein-coding genes and creating the Volcano plot:

data1Prot <- merged1[str_detect(merged1$biotype, "protein_coding"), ]

plot1prot <- ggplot(data1Prot, aes(x = log_fold_change, y = log_p_values)) + geom_point(aes(color = Significant)) +scale_x_continuous(limits = c(-1.3, 1.3),expand=c(0,0)) + scale_y_continuous(limits=c(0,4.6),expand=c(0,0)) + scale_color_manual(values = c("gray", "red")) + ggtitle("DU Protein coding genes in RTX_UGI vs 5FdUR_UGI") + theme_light() + geom_rect(aes(xmin=-0.58496,xmax=0.58496,ymin=1.30103,ymax=Inf),alpha=0.02,fill="grey99") + geom_rect(aes(xmin=-Inf,xmax=Inf,ymin=0,ymax=1.30103),alpha=0.02,fill="grey99") + theme(text = element_text(size=20))

ggsave("plot1prot.png", plot1prot, dpi = 600)

#Selection of immunoglobulin and T-cell receptor genes and creating the Volcano plot:

data1IG <- merged1[str_detect(merged1$biotype, "IG"), ]

data1TR <- merged1[str_detect(merged1$biotype, "TR"), ]

data1IGtr <- rbind(data1TR, data1IG)

plot1IGTR <- ggplot(data1IGtr, aes(x = log_fold_change, y = log_p_values)) + geom_point(aes(color = Significant)) +scale_x_continuous(limits = c(-1.3, 1.3),expand=c(0,0)) + scale_y_continuous(limits=c(0,4.6),expand=c(0,0)) + scale_color_manual(values = c("gray", "red")) + ggtitle("DU IG and TR genes in RTX_UGI vs 5FdUR_UGI") + theme_light() + geom_rect(aes(xmin=-0.58496,xmax=0.58496,ymin=1.30103,ymax=Inf),alpha=0.02,fill="grey99") + geom_rect(aes(xmin=-Inf,xmax=Inf,ymin=0,ymax=1.30103),alpha=0.02,fill="grey99") + theme(text = element_text(size=20))

ggsave("plot1IGTR.png", plot1IGTR, dpi = 600)

#Selection of small RNA genes and creating the Volcano plot:

data1sno <- merged1[str_detect(merged1$biotype, "snoRNA"), ]

data1sn <- merged1[str_detect(merged1$biotype, "snRNA"), ]

data1s <- merged1[str_detect(merged1$biotype, "sRNA"), ]

data1sRNAs <- rbind(data1sno, data1sn, data1s)

plot1sRNAs <- ggplot(data1sRNAs, aes(x = log_fold_change, y = log_p_values)) + geom_point(aes(color = Significant)) +scale_x_continuous(limits = c(-1.3, 1.3),expand=c(0,0)) + scale_y_continuous(limits=c(0,4.6),expand=c(0,0)) + scale_color_manual(values = c("gray", "red")) + ggtitle("DU sRNA genes in RTX_UGI vs 5FdUR_UGI") + theme_light() + geom_rect(aes(xmin=-0.58496,xmax=0.58496,ymin=1.30103,ymax=Inf),alpha=0.02,fill="grey99") + geom_rect(aes(xmin=-Inf,xmax=Inf,ymin=0,ymax=1.30103),alpha=0.02,fill="grey99") + theme(text = element_text(size=20))

ggsave("plot1sRNAs.png", plot1sRNAs, dpi = 600)

#Selection of long non-coding RNA genes and creating the Volcano plot:

data1lnc <- merged1[str_detect(merged1$biotype, "lncRNA"), ]

plot1lncRNAs <- ggplot(data1lnc, aes(x = log_fold_change, y = log_p_values)) + geom_point(aes(color = Significant)) +scale_x_continuous(limits = c(-1.3, 1.3),expand=c(0,0)) + scale_y_continuous(limits=c(0,4.6),expand=c(0,0)) + scale_color_manual(values = c("gray", "red")) + ggtitle("DU lncRNA genes in RTX_UGI vs 5FdUR_UGI") + theme_light() + geom_rect(aes(xmin=-0.58496,xmax=0.58496,ymin=1.30103,ymax=Inf),alpha=0.02,fill="grey99") + geom_rect(aes(xmin=-Inf,xmax=Inf,ymin=0,ymax=1.30103),alpha=0.02,fill="grey99") + theme(text = element_text(size=20))

ggsave("plot1lncRNAs.png", plot1lncRNAs, dpi = 600)

#Selection of microRNA genes and creating the Volcano plot:

data1miR <- merged1[str_detect(merged1$biotype, "miRNA"), ]

plot1miRNAs <- ggplot(data1miR, aes(x = log_fold_change, y = log_p_values)) + geom_point(aes(color = Significant)) +scale_x_continuous(limits = c(-1.3, 1.3),expand=c(0,0)) + scale_y_continuous(limits=c(0,4.6),expand=c(0,0)) + scale_color_manual(values = c("gray", "red")) + ggtitle("DU miRNA genes in RTX_UGI vs 5FdUR_UGI") + theme_light() + geom_rect(aes(xmin=-0.58496,xmax=0.58496,ymin=1.30103,ymax=Inf),alpha=0.02,fill="grey99") + geom_rect(aes(xmin=-Inf,xmax=Inf,ymin=0,ymax=1.30103),alpha=0.02,fill="grey99") + theme(text = element_text(size=20))

ggsave("plot1miRNAs.png", plot1miRNAs, dpi = 600)

**#Variant calling using Mutect2 of GATK4 package.** Variants in the tested samples (cf. Table 5) are called against a control termed “normal” in Mutect2.

gatk Mutect2 -R GRCh38.d1.vd1.fa -I TEST_NAME.bam -I WT_NAME.bam -normal WT_GROUP_NAME -O TEST_NAME_vs_WT_NAME.vcf

#The raw variants were annotated by GATK’s FilterMutectCalls by the following script:

gatk FilterMutectCalls -R GRCh38.d1.vd1.fa -V TEST_NAME_vs_WT_NAME.vcf -O TEST_NAME_vs_WT_NAME_FILTERED.vcf

**#Calculation and analysis of mutational profiles (Fig 4A, Fig 7A, and Fig S5A).** The six types of SBSs were derived and filtered from the output VCF files. Their occurrence (sum of the counts weighted with their allele frequencies), and their frequencies (occurrence of one SBS / occurrence of all types of SBSs) were calculated.

from pathlib import Path

import pandas as pd

def read_vcf(vcf_file_path: Path):

"""read and filter vcfs"""

original_dataframe = pd.read_csv(vcf_file_path, sep="\t",

comment="#", header=None,

names=["#CHROM", "POS", "ID", "REF", "ALT", "QUAL",

"FILTER", "INFO", "FORMAT",

"NON_TREATED", "uracil_seq"])

filtered_dataframe = original_dataframe[~(

(original_dataframe["FILTER"].str.contains("weak_evidence"))

| (original_dataframe["FILTER"].str.contains("normal_artifact"))

| (original_dataframe["FILTER"].str.contains("slippage"))

| (original_dataframe["FILTER"].str.contains("germline"))

| (original_dataframe["FILTER"].str.contains("map_qual"))

| (original_dataframe["FILTER"].str.contains("position"))

| (original_dataframe["FILTER"].str.contains("base_qual")))]

return filtered_dataframe

def compute_sbs_allelic_frequency(filtered_dataframe: pd.DataFrame):

"""compute all sbs afs"""

df_all_sbs = filtered_dataframe[(filtered_dataframe["REF"].isin(["C", "T", "G", "A"]))

& (filtered_dataframe["ALT"].isin(["C", "T", "G", "A"]))]

afs = df_all_sbs["uracil_seq"].str.split(":").str[2].astype(float)

return afs.sum()

def compute_transition_allelic_frequency(filtered_dataframe: pd.DataFrame, ref_1: str,

alt_1: str, ref_2: str, alt_2: str):

"""compute specific sbs afs"""

df_specific_sbs = filtered_dataframe[((filtered_dataframe["REF"] == ref_1)

& (filtered_dataframe["ALT"] == alt_1))

| ((filtered_dataframe["REF"] == ref_2)

& (filtered_dataframe["ALT"] == alt_2))]

afs = df_specific_sbs["uracil_seq"].str.split(":").str[2].astype(float)

return afs.sum()

if __name__ == "__main__":

files = Path.cwd().glob("*.vcf")

out_df = pd.DataFrame(index=["all_sbs", "ctot", "ctoa", "ctog", "ttoa", "ttoc", "ttog"])

for file in files:

filtered_df = read_vcf(file)

all_sbs = compute_sbs_allelic_frequency(filtered_df)

ctot = compute_transition_allelic_frequency(filtered_df,

ref_1="C", alt_1="T", ref_2="G", alt_2="A")

ctoa = compute_transition_allelic_frequency(filtered_df,

ref_1="C", alt_1="A", ref_2="G", alt_2="T")

ctog = compute_transition_allelic_frequency(filtered_df,

ref_1="C", alt_1="G", ref_2="G", alt_2="C")

ttoa = compute_transition_allelic_frequency(filtered_df,

ref_1="T", alt_1="A", ref_2="A", alt_2="T")

ttoc = compute_transition_allelic_frequency(filtered_df,

ref_1="T", alt_1="C", ref_2="A", alt_2="G")

ttog = compute_transition_allelic_frequency(filtered_df,

ref_1="T", alt_1="G", ref_2="A", alt_2="C")

result = [all_sbs, ctot, ctoa, ctog, ttoa, ttoc, ttog]

print(result)

out_df[str(file.stem)] = result

out_df_= out_df.transpose()

out_df_.to_csv(str(Path.cwd().stem) + ".csv", sep="\t")

#For statistical evaluation, the 7 variation calling experiments for each comparison (cf. Source data file 8) were tested by Welch’s two-sample t-tests.

import numpy as np

from scipy import stats

# Sample data

NTvsWT = np.array([0.374, 0.360, 0.365, 0.363, 0.376, 0.377, 0.369])  # Sample 1

FUvsWT = np.array([0.478, 0.597, 0.529, 0.565, 0.477, 0.412, 0.429])  # Sample 2

# Welch's t-test

t_stat, p_value = stats.ttest_ind(NTvsWT, FUvsWT, equal_var=False)

# Means and standard deviations

mean1, mean2 = np.mean(NTvsWT), np.mean(FUvsWT)

std1, std2 = np.std(NTvsWT, ddof=1), np.std(FUvsWT, ddof=1)

# Sample sizes

n1, n2 = len(NTvsWT), len(FUvsWT)

# Calculate effect size (Cohen's d for Welch's t-test)

# Pooled standard deviation for Welch's t-test

pooled_std = np.sqrt(((n1 - 1) * std1**2 + (n2 - 1) * std2**2) / (n1 + n2 - 2))

cohens_d = (mean1 - mean2) / pooled_std

# Output

print(f"T-statistic: {t_stat}")

print(f"P-value: {p_value}")

print(f"Effect size (Cohen's d): {cohens_d}")

**#Calculate the occurrence of variants according to replication timing (Fig 5A)**

#The replication timing data were split into 3 classes (EARLY, MIDDLE, LATE).

bedtools sort -i hct_116_rt_rep1_rep2.bed > hct_116_rt_rep1_rep2_sorted.bed

bedtools merge -i hct_116_rt_rep1_rep2_sorted.bed -c 4 -o mean >rep_timing.bed

#The Python Bioframe module was used to calculate the overlap between variants and replication timing data:

import pandas as pd

import bioframe as bf

colnames = ["CHROM","START","END","LABEL"]

df_filtered_all_sbs = pd.read_csv("filtered.vcf")

rep_times = pd.read_csv("rep_timing.bed",sep="\t")

df_filtered_all_sbs ["AF_SBS"] = df_filtered_all_sbs ["TUMOR_NAME"].str.split(":").str[2].astype(float)

df_filtered_all_sbs_[“end”] = df_filtered_all_sbs["POS"] + 1

overlapping_intervals = bf.overlap(rep_times,df_filtered_all_sbs,how="left",cols1=("CHROM","START","END"),cols2=("CHROM","POS","end"))[["CHROM","START","END","LABEL","AF_SBS_"]]

overlapping_intervals_grouped = overlapping_intervals.groupby(["CHROM","START","END","LABEL"],as_index=False).sum()

result = overlapping_intervals_grouped.groupby("LABEL",as_index=False)["AF_SBS_"].sum()

#This procedure was repeated for only C-to-T transitions, and a ratio was calculated.

**#Calculate the occurrence of C-to-T transitions for the genomic segments of uracilation (Fig 5B and Fig S7).** The Python Bioframe module was used to calculate the overlap between variants (either all SBSs or only the C-to-T transitions in VCF file) and the genomic segments (BED file).

import pandas as pd

import bioframe as bf

colnames = ["CHROM","START","END","LABEL","INFO1","INFO2","INFO3","INFO4","INFO5"]

segments = pd.read_csv("segments.bed",sep="\t", names = colnames )

df_filtered_all_sbs ["AF_SBS"] = df_filtered_all_sbs ["TUMOR_NAME"].str.split(":").str[2].astype(float)

df_filtered_all_sbs_["end"] = df_filtered_all_sbs["POS"] + 1

overlapping_intervals = bf.overlap(segments,df_filtered_all_sbs,how="left",cols1=("CHROM","START","END"),cols2=("CHROM","POS","end"))[["CHROM","START","END","LABEL","AF_SBS_"]]

overlapping_intervals_grouped = overlapping_intervals.groupby(["CHROM","START","END","LABEL"],as_index=False).sum()

results = overlapping_intervals_grouped.groupby("LABEL",as_index=False)["AF_SBS_"].sum()

#This procedure was repeated for only CtoT transitions, and a ratio was calculated.

**#Computing mutational spectra (Fig 4B, Fig 7B, Fig S4, Fig S5B, FigS6, and Fig S10).** using SigProfiler Matrix Generator 1.1.21 and SigProfiler Plotting Python modules.

from SigProfilerMatrixGenerator import install as genInstall

genInstall.install('GRCh38', rsync=False, bash=True)

from SigProfilerMatrixGenerator.scripts import SigProfilerMatrixGeneratorFunc as matGen

matrices = matGen.SigProfilerMatrixGeneratorFunc("TEST_NAME", "GRCh38", path to "TEST_NAME_WITH_WT_NAME")

#Plotting of all "96", "78" and "1536" type matrices.

import sigProfilerPlotting as sigPlt

sigPlt.plotSBS(NAME.SBS96.all, output_path, project, 96, percentage=False)

sigPlt.plotSBS(NAME.DBS78.all, output_path, project, 78, percentage=False)

sigPlt.plotSBS(NAME.SBS1535.all, output_path, project, 1535, percentage=False)

**#Analysis of potential stem-loop structure around the detected C-to-T transitions (Fig S11C)**. Extraction of surrounding sequences +/- 15 nt around the detected C-to-T transitions; both clustered and non-clustered ones

for f in ./*UGI_*.csv

do

tsamp="$(basename $f .csv)"

awk '($4~/clustered/) && (($0~/CtoT/) || ($0~/GtoA/)) {print $2 /

"\t" ($3-16) "\t" ($3+15)}' $tsamp.csv > $RESULTS/$tsamp.clustered_CtoT.bed

awk '($0!~/CtoT/) && ($0!~/GtoA/) && ($4~/clustered/) {print $2 "\t" /

($3-16) "\t" ($3+15)}' $tsamp.csv > $RESULTS/$tsamp.other_clustered_SBS.bed

awk '($4~/PASS/) && (($0~/CtoT/) || ($0~/GtoA/)) {print $2 "\t" ($3-16) /

"\t" $3+15)}' $tsamp.csv > $RESULTS/$tsamp.PASS_CtoT.bed

awk '($0!~/CtoT/) && ($0!~/GtoA/) && ($4~/PASS/) {print $2 "\t" ($3-16) /

"\t" ($3+15)}' $tsamp.csv > $RESULTS/$tsamp.other_PASS_SBS.bed

Done

#Extracting the reference sequences to the bed files using bedtools nuc:

for f in ./*.bed

do

tsamp="$(basename $f .bed)"

bedtools nuc -s -seq -fi $PATH/GRCh38.d1.vd1.fa -bed $tsamp.bed | awk /

'{print $1 "\t" ($2+16) "\t" $13}' > $tsamp.seq.csv

done

#Python code for the prediction of stem-loop structures in the sequences defined in the bed files:

import csv

import os

from collections import Counter, defaultdict

def inverted_complement(s):

return s.translate(str.maketrans("ATCGNatcgn", "TAGCNtagcn"))[::-1]

def find_best_hairpin(main_dir, file, seq, variant_pos, window=15, min_stem=4, loop_min=3, loop_max=6):

seq_len = len(seq)

best_hairpin = None

best_stem_len = 0

variant_nucleotide_type = seq[variant_pos]

final_variant_positions_in_loop = {}

for i in range(seq_len): #iterate over sequence

max_possible_stem = (seq_len - i) // 2 #max possible stem length

stem_len = max_possible_stem

while stem_len >= min_stem:

left = seq[i:i + stem_len]

for loop_len in range(loop_min, loop_max + 1):

loop_start = i + stem_len

loop_end = loop_start + loop_len

right_start = loop_end

right_end = right_start + stem_len

if right_end > seq_len:

continue

right = seq[right_start:right_end]

if inverted_complement(left) == right:

if i <= variant_pos < right_end:

rel_pos = variant_pos - i

left_part, loop_part, right_part = left, seq[loop_start:loop_end], right

variant_positions_in_loop = {}

if rel_pos < stem_len:

left_part = left[:rel_pos] + "*" + left[rel_pos + 1:]

elif rel_pos < stem_len + loop_len: #variant in loop

loop_rel = variant_pos - loop_start

if "CtoT" in file and variant_nucleotide_type.upper() == "C":

pos_in_loop = loop_rel + 1 # 1-based, left-to-right

elif "CtoT" in file and variant_nucleotide_type.upper() == "G":

pos_in_loop = loop_len - loop_rel # 1-based, right-to-left

else:

pos_in_loop = loop_rel + 1

variant_positions_in_loop.setdefault(loop_len, []).append(pos_in_loop)

loop_part = loop_part[:loop_rel] + "*" + loop_part[loop_rel + 1:]

else:

right_rel = variant_pos - loop_end

right_part = right[:right_rel] + "*" + right[right_rel + 1:]

formatted = f"{left_part}[{loop_part}]{right_part}" #formatted string * marks variant

if stem_len > best_stem_len:

best_hairpin = formatted

best_stem_len = stem_len

final_variant_positions_in_loop = variant_positions_in_loop

stem_len -= 1

if best_hairpin:

with open(os.path.join(main_dir, "raw", f"{file}_found_hairpins.txt"), "a") as d:

if len(final_variant_positions_in_loop) == 0:

d.write(f"{len(loop_part)} \t\t{best_hairpin}\t{variant_nucleotide_type}\n")

else:

for loop_len, positions in final_variant_positions_in_loop.items():

for pos in positions:

d.write(f"{loop_len}\t{pos}\t{best_hairpin}\t{variant_nucleotide_type}\n")

return [best_hairpin], final_variant_positions_in_loop, variant_nucleotide_type

else:

return [], {}, variant_nucleotide_type

def process_batch(main_dir, file, seq_list, variant_positions):

all_hairpins = []

loop_variant_total = 0

loop_position_counts = Counter()

variant_nts = []

for seq in seq_list:

hairpins, positions_dict, variant_nt = find_best_hairpin(main_dir, file, seq, variant_positions)

all_hairpins.extend(hairpins)

if len(positions_dict) > 0:

loop_variant_total += 1

variant_nts.append(variant_nt)

for loop_len, positions in positions_dict.items():

for pos in positions:

loop_position_counts[(loop_len, pos)] += 1

return all_hairpins, loop_variant_total, loop_position_counts, variant_nts

def write_summary(main_dir, file, row_counter, seq_list, variant_positions):

hairpins, loop_variant_total, loop_position_counts, variant_nts = process_batch(main_dir, file, seq_list, variant_positions)

with open("final_result.txt", "a") as f:

f.write(f"{file}\t{row_counter}\t{len(hairpins)}\t{loop_variant_total}\t")

for (loop_len, pos), count in sorted(loop_position_counts.items()):

f.write(f"{count}\t")

f.write("\n")

def main():

main_dir = "" # set your dir

for file in os.listdir(main_dir):

if not os.path.isdir(file):

with open (os.path.join(main_dir, file)) as seq:

print(os.path.join(main_dir, file), "is being processed")

seq_list = []

variant_position = 15 #31 nt seqs

tsv_reader = csv.reader(seq, delimiter="\t")

next(tsv_reader) #header skip

row_counter =0

for row in tsv_reader:

(chrom, pos, seq ) = row

seq_list.append(seq)

row_counter += 1

write_summary(main_dir, file, row_counter, seq_list=seq_list, variant_positions=variant_position)

if __name__ == "__main__":

main()

**#The inter-mutational distances (Fig 4C)** were calculated using awk.

awk '{if(NR>1 && _k ==$1 ) {print $2-_n};_n=$2;_k=$1}' NAME.filteredSBS.vcf > NAME.dist

#The log histograms were made using ggplot2 in R.

X <- read.delim("~/PATH/NAME.dist")

p<-ggplot(X, aes(x=dist)) +  geom_histogram(color="black", fill="white",binwidth=0.15) + theme_light() + scale_y_continuous(expand=c(0,0),limits=c(0,1550)) + scale_x_continuous(expand=c(0,0),limits=c(0,8))

**#R-script for the box plot in Fig 6A**

>install.packages("ggplot2")

>install.packages("patchwork")

>library(ggplot2)

>library(patchwork)

>cellCycleSumma <- read.delim("~/PATH/source_data_file_12.csv", row.names="No")

>View(cellCycleSumma)

>group_order <- c("NT", "RTX_0.1", "RTX_1", "RTX_20", "5FdUR_0.1", "5FdUR_1", "5FdUR_20")

>cellCycleSumma$label <- factor(cellCycleSumma$label, levels = group_order)

>G1G0 <- ggplot(cellCycleSumma, aes(x = label, y = G1)) + geom_boxplot(width = 0.65, fill = alpha("black", 0.3), color = "black", outlier.shape = NA) + geom_point(position = position_jitter(width = 0.3), color = "black", alpha = 1, size = 2) + scale_y_continuous(limits = c(0, 60),expand = c(0, 0)) + labs(title = "G0/G1 phase", x = NULL, y = "Percentage of living cells") + theme_light() + theme(text = element_text(size=12), plot.title = element_text(hjust = 0.5, vjust = 0), axis.text.x = element_text(angle = 90, hjust = 1, vjust = 0))

>ggsave("~/PATH/G1G0cr.png", plot = G1G0, width = 4.35, height = 5.79, dpi = 600)

>Sphase <- ggplot(cellCycleSumma, aes(x = label, y = S)) + geom_boxplot(width = 0.65, fill = alpha(rgb(0.502, 0.392, 0.635), 0.3), color = rgb(0.502, 0.392, 0.635), outlier.shape = NA) + geom_point(position = position_jitter(width = 0.3), color = rgb(0.502, 0.392, 0.635), alpha = 1, size = 2) + scale_y_continuous(limits = c(0, 100),expand = c(0, 0)) + labs(title = "S phase", x = NULL, y = NULL) + theme_light() + theme(text = element_text(size=12), plot.title = element_text(hjust = 0.5, vjust = 0), axis.text.x = element_text(angle = 90, hjust = 1, vjust = 0))

>ggsave("~/PATH/SphaseCr.png", plot = Sphase, width = 4.35, height = 5.79, dpi = 600)

>G2M <- ggplot(cellCycleSumma, aes(x = label, y = M)) + geom_boxplot(width = 0.65, fill = alpha(rgb(0.576, 0.537, 0.325), 0.3), color = rgb(0.576, 0.537, 0.325), outlier.shape = NA) + geom_point(position = position_jitter(width = 0.3), color = rgb(0.576, 0.537, 0.325), alpha = 1, size = 2) + scale_y_continuous(limits = c(0, 25),expand = c(0, 0)) + labs(title = "G2/M phase", x = NULL, y = NULL) + theme_light() + theme(text = element_text(size=12), plot.title = element_text(hjust = 0.5, vjust = 0), axis.text.x = element_text(angle = 90, hjust = 1, vjust = 0))

>ggsave("~/PATH/G2Mcr.png", plot = G2M, width = 4.35, height = 5.79, dpi = 600)

>combined_plot <- G1G0 + Sphase + G2M

>ggsave("~/PATH/combined_plotcr.png", combined_plot, width = 8, height = 4, dpi = 600)
