# Supplementary material for: Effects of thymidylate synthase inhibitors differ in genomic uracilation and mutagenic potential
Source: Life Sci Alliance. 2026 Feb 6;9(4):e202503352. doi: 10.26508/lsa.202503352 (PMC12881662; doi:10.26508/lsa.202503352)
Supplement: Supplementary file 6 [file LSA-2025-03352_TableS1.docx]

| sample | Uracil content*  (uracil / million bases) | Number of uracil within the genome (total) |
| --- | --- | --- |
| WT | n. d. | 3540 |
| NT_UGI | 1.28 +/- 0.16 | 3540 |
| NT_UGI_MMR | 1.99 +/-0.23 | 5504 |
| 5FdUR_UGI | 388 +/- 35 | 1072388 |
| RTX_UGI | 685 +/- 35 | 1894353 |
| 5FdUR_UGI_MMR | 892 +/-31 | 2467168 |
| RTX_UGI_MMR | 961 +/-24 | 2658014 |

***Supplementary Table 1. Global uracil content as determined by dot blot measurements in (Palinkas et al, 2020).***
