# Supplementary material for: Effects of thymidylate synthase inhibitors differ in genomic uracilation and mutagenic potential
Source: Life Sci Alliance. 2026 Feb 6;9(4):e202503352. doi: 10.26508/lsa.202503352 (PMC12881662; doi:10.26508/lsa.202503352)
Supplement: Supplementary file 19 [file LSA-2025-03352_SdataF8.2.pdf]

Source Data File 17 – Uncropped gel image for Fig. 8C.

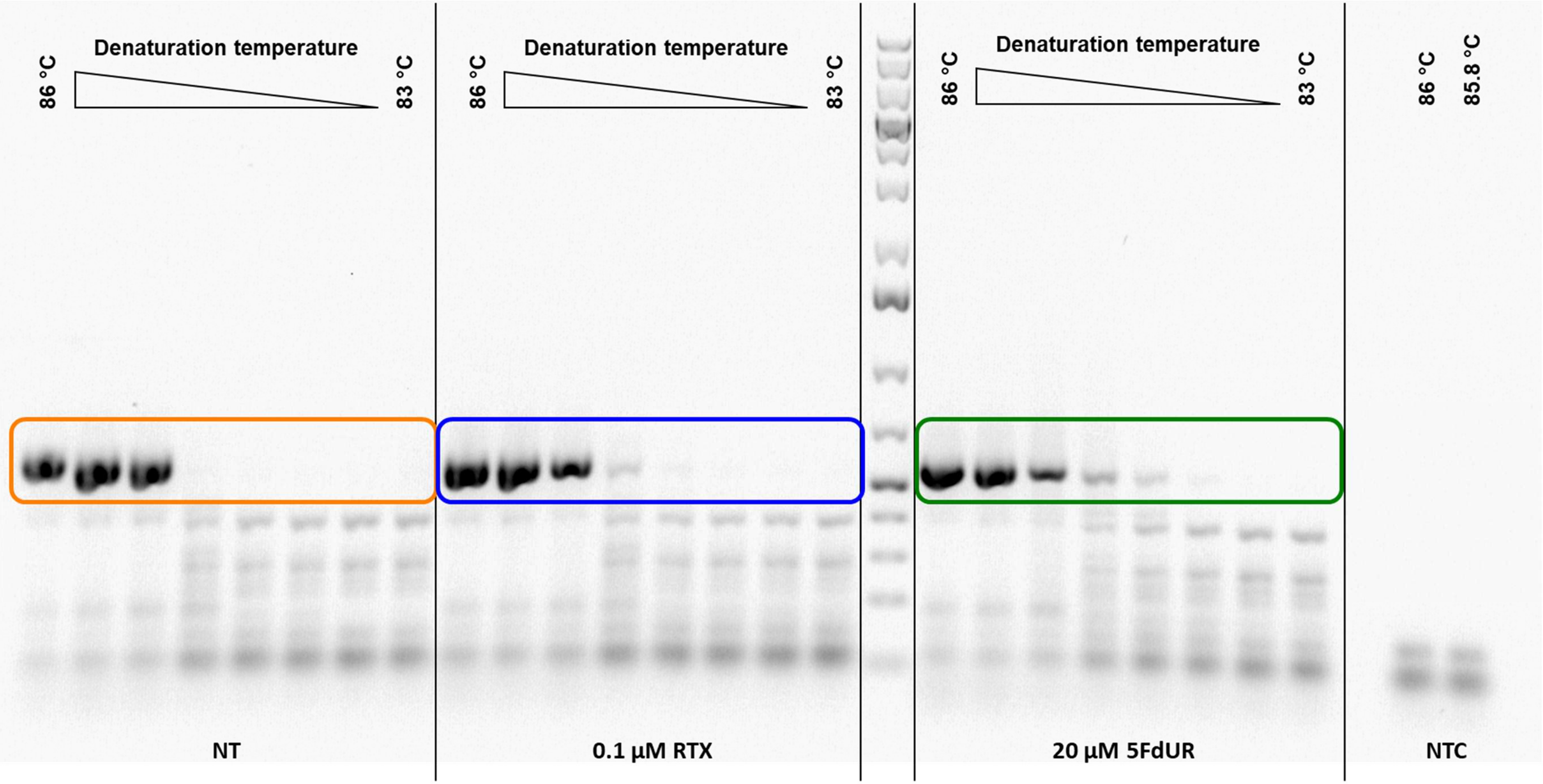

Agarose gel of 3D-PCR targeting the MDM2 genomic region. Samples: 3D-PCR products non-treated (NT), 0.1 µM RTX-treated (0.1 µM RTX), and 20 µM 5FdUR-treated (20 µM 5FdUR) UNG-inhibited HCT116 cells. The expected PCR product size is 570 bp. GeneRuler 1 kb Plus DNA Ladder (Thermo Fisher Scientific) was used as the molecular size marker. The differential denaturation temperatures are indicated above the gel. No-template controls (NTC) corresponding to the two highest denaturation temperatures are shown on the right. Colored boxes highlight the gel sections displayed in Fig 8C.
