# Supplementary material for: Effects of thymidylate synthase inhibitors differ in genomic uracilation and mutagenic potential
Source: Life Sci Alliance. 2026 Feb 6;9(4):e202503352. doi: 10.26508/lsa.202503352 (PMC12881662; doi:10.26508/lsa.202503352)
Supplement: Supplementary file 23 [file LSA-2025-03352_TableS2.docx]

|  | **control** | **test** | **event** | **detected variant** |
| --- | --- | --- | --- | --- |
| *original CpG* | CG | CG | - | - |
|  |  | TG | C->T | C->T |
|  |  | CA | G->A | C->T |
|  |  | TA | CG->TA | **CG->TA** |
| *asymmetric deamination* | TG | CG | T->C | T->C |
|  |  | TG | - | - |
|  |  | CA | TG->CA | **TG->CA** |
|  |  | TA | G->A | C->T |
| *asymmetric deamination* | CA | CG | A->G | T->C |
|  |  | TG | CA->TG | **TG->CA** |
|  |  | CA | - | - |
|  |  | TA | C->T | C->T |
| *symmetric deamination* | TA | CG | TA->CG | **TA->CG** |
|  |  | TG | A->G | T->C |
|  |  | CA | T->C | T->C |
|  |  | TA | - | - |

***Supplementary Table 2****.* ***Considerations for possible DBSs and SBSs that are related to a background CpG deamination appearing either in the control or in the test samples.***
